# Endothelial Cell-Derived TGF-β Promotes Epithelial-Mesenchymal Transition via CD133 in HBx-Infected Hepatoma Cells

**DOI:** 10.3389/fonc.2019.00308

**Published:** 2019-04-24

**Authors:** Preety Rawal, Hamda Siddiqui, Mohsin Hassan, Manish Chandra Choudhary, Dinesh M. Tripathi, Vikrant Nain, Nirupama Trehanpati, Savneet Kaur

**Affiliations:** ^1^School of Biotechnology, Gautam Buddha University, Greater Noida, India; ^2^Department of Molecular and Cellular Medicine, Institute of Liver and Biliary Sciences, New Delhi, India

**Keywords:** epithelial to mesenchymal transition (EMT), endothelial cells, hepatocellular carcinoma (HCC), hepatitis B virus X protein (HBx), transforming growth factor Beta (TGF-β), tumor microenvironment

## Abstract

**Background:** Hepatitis B-X Protein (HBx) encoded in Hepatitis B virus (HBV) is known to play a critical role in development and progression of HBV induced hepatocellular carcinoma (HCC). HBx interacts with and activates various cells in HCC microenvironment to promote tumor initiation, progression and invasion. In this study, we investigated how surrounding stromal cells interact with HBx-infected hepatoma cells by a series of *in vitro* co-culture studies.

**Methods:** Huh7 hepatoma cells were cultured and transfected with the mammalian expression vector pGFP-HBx. Co-culture assays were performed between HBx-transfected Huh7 cells and conditioned media (CM) from stromal cells [endothelial cell lines (HUVECs) and hepatic stellate cell lines (LX2 cells)]. The effect of these interactions was studied by a series of functional assays like chemotaxis, invasion, and wound healing scratch assays. Also, quantitative real time (RT)-PCRs of the mesenchymal genes was performed in the hepatoma cells with and without the co-cultures. Hep3B cells with an integrated HBV genome were taken as positive controls.

**Results:** HBx-transfected Huh7 cells cultured in presence of CM from HUVECs illustrated enhanced migration and tube formation as compared to HBx-transfected cells cultured alone or co-cultured with LX2 cells. HBx-transfected hepatoma cells incubated with CM from HUVECs also expressed mesenchymal genes including Thy1, CDH2, TGFβR1, VIM, and CD133. ELISAs revealed increased levels of TGF-β in CM from HUVECs. In comparison to unstimulated HBx-transfected Huh7 cells, TGF-β stimulated cells displayed increased invasive properties and mesenchymal gene expression. RT-PCR and flow cytometry analysis further demonstrated that incubation with either CM from HUVECs or TGF-β significantly increased the expression of a stemness marker, CD133 in HBx-infected hepatoma cells. Gene inhibition experiments with CD133 siRNA showed a downregulation of mesenchymal gene expression and properties in TGF-β induced HBx-infected hepatoma cells as compared to that observed in control siRNA treated cells, indicating CD133 as one of the key molecules affecting epithelial to mesenchymal transition (EMT) in HBx-infected cells.

**Conclusion:** The study indicates that secretory factors like TGF-β from neighboring endothelial cells may enhance expression of CD133 and impart an aggressive EMT phenotype to HBx-infected hepatoma cells in HBV induced HCC.

## Introduction

Hepatocellular Carcinoma (HCC) is one of the most common cancer worldwide, representing approximately 4% of all malignancies (1). It has been estimated that more than 50% of HCC cases in the world are associated with hepatitis B virus (HBV) (2). HBV is a partially double stranded DNA virus belonging to the Hepadnavirus family. The HBV genome is 3.2 kb in size and contains four overlapping major open reading frames tightly arranged that encode polymerase, surface (HBsAg), core (HBcAg), and X proteins (HBx). HBx, a 17-kd protein is the most frequently integrated viral sequence found in HBV-induced HCCs (3). HBx is known to interact with various transcription factors of the host and affect activation and modulation of several signal transduction pathways (4). The activation of these signal transduction pathways by HBx leads to the upregulation of a number of cellular genes, including those of growth factors and oncogenes. During late stages of tumor progression, HBx also drives the activation of cellular pathways associated with metastasis and angiogenesis, which play an important role in the growth and spread of HCC (5).

Many recent studies have now demonstrated that the growth and spread of tumor is not only affected by alterations in the tumor cells themselves but also by the surrounding niche cells (6). Microenvironment components are known to both inhibit and augment the activity of the tumor cells (7). The stromal environment of HCC in liver consists of several cell types, endothelial cells, hepatic stellate cells, macrophages and immune cells. Studies have reported that HBx interacts and activates most of these cells in liver tumor microenvironment to promote tumor initiation, progression, invasion, and metastasis (8). In the current study, we investigated the effects of two types of stromal cells, that is, endothelial cells and hepatic stellate cells on tumor properties of HBx-infected hepatoma cells.

## Materials and Methods

### Cell Cultures

Huh7, HepG2, and Hep3B cells were cultured in Dulbecco's Modified Eagle's medium (DMEM) (GIBCO) with 10% FBS (Hyclone) and 100 μg/ml streptomycin and 100 IU/ml penicillin (GIBCO) at 37°C in humified atmosphere containing 5% CO_2_. LX2 cells (Hepatic stellate cells) were cultured in Dulbecco's Modified Eagle's medium (DMEM) (Gibco) with 2% FBS (Hyclone) and 100 μg/ml streptomycin and 100 IU/ml penicillin at 37°C with 5% CO_2_. HUVECs cells [Human umbilical venules endothelial cells (GIBCO), purchased from Invitrogen] were grown in Endothelial medium (HiMedia Laboratories) with growth factors and 1% antibiotics on gelatin coated plates.

#### Coculture of Cells

To study how endothelial cells and stellate cells modulate the tumorigenic behavior of HBx-Huh7, we performed the assays in indirect and direct co-cultures. For indirect co-cultures, Huh7 cells were treated with conditioned media (CM) from HUVECs/LX2 cells. CMs were prepared after serum starvation of these cells for 24 h and then collecting the supernatants after centrifugation to remove cell debris. For direct co-cultures, Huh7 cells were mixed with LX2/HUVECs in similar ratios to create 1:1 coculture system. For obtaining TGF-β treated cells, hepatoma cells were exposed to 5 ng/ml concentration as depicted by ELISA used for 48 h (9, 10).

### Transfection in Hepatoma Cell Lines

Huh7 and HepG2 cells were transfected with the mammalian expression vector pGFP-HBx (purchased from addgene # 24931) (11). The vector was amplified in *Escherichia coli* cells, followed by plasmid isolation using the plasmid isolation kit (Promega, India). For transfection, lipofectamine 2000 (ThermoFisher Scientific, Invitrogen #11668-019) was used according to manufacturer's instructions. As a control, pcDNA3-EGFP plasmid vector (kind gift from Dr. Vijay) was used as control in all transfection experiments. Huh7 and Hep3B cells were further silenced by transfection with CD133 siRNA (purchased from ThermoFisher Scientific #AM16708) and control siRNA (addgene #10900)′ using Lipofectamine reagent 2000 as per the instructions. Forty-eight hours after transfection, the cells were seen under an inverted fluorescent microscope (Nikon ECLIPSE Ti).

### Chemotaxis and Invasion Assays

HBx-transfected, control-transfected, CD133 silenced and TGF-β stimulated hepatoma cells were detached, harvested by centrifugation and resuspended in DMEM (without serum), and then placed in the upper chamber of a modified Boyden chamber consisting of uncoated polycarbonate filter membranes of 8 μm pore size. For invasion assays, transwell insert first coated with matrigel.The chamber was placed in a 24-well culture dish containing DMEM (as control), LX2 and HUVECs cells as monolayer (50,000 cells/well seeded overnight prior to experiment) in lower chamber. For chemotaxis, after 24 h incubation and for invasion, after 48 h, at 37°C, the lower side of the filter was washed with PBS and fixed with 4% paraformaldeyde for 2 min. Then cells were washed and permeabilized by 100% methanol for 20 min. For quantification, cell nuclei were stained with 0.5% crystal violet. The upper side of the filter containing the non-migrating cells was scraped with a cotton swab. Cells migrating toward the lower chamber were counted manually at 4X objective in random microscopic fields.

### Wound Healing/Scratch Migration Assays

HBx-transfected, control-transfected, CD133 silenced and TGF-β stimulated hepatoma cells were plated in 12-well plates (3 × 10^6^cells/well). After 6 h of serum starved condition, a scratch was made on the cell layer using a 100 μl sterile micropipette tip to create a wound. Cellular debris was carefully removed by washing with media to remove floating cells. The CM from LX2 and HUVECs were added to the cells and incubated for the next 24 h (as indirect cocultures). The cells were photographed using a phase-contrast microscope, to determine the wound width at time 0 h. The cultures were continued, and the cells were photographed again after 24 h of wounding the cell layer. Wound healing was visualized by comparing photographs taken at 0 h with 24 h later and analyzed for the distance migrated by the leading edge of the wound at each time point in all the study groups. The relative wound width was measured as wound width at the time 24 h divided by wound width at time point 0 h. The measurements were done by Software NIS Elements from NIKON Eclipse Ti. The unit for measurement was μm.

### Quantitative RT-PCR (qRT-PCR) Analysis

For qRT-PCR, cells were harvested by using trypsin-EDTA solution (0.25%). Total RNA was isolated by using Nucleopore kit as per manufacturer's instructions. RNA quantified at 260/280 nm with Thermo Scientific Nanodrop 2000 Spectrophotometer. The absorption ratio A260 nm/A280 nm between 1.90 and 2 was taken into consideration for cDNA preparation. First strand cDNA was synthesized from 1 μg of total RNA with reverse transcriptase (Thermo Scientific Verso cDNA synthesis kit) according to manufacturer instructions. Quantitative real time PCR was carried with SYBR green PCR master mix (Fermentas Life Sciences) on the ViiA7 instrument PCR system (Applied Biosystems, USA). Dissociation curve was generated at the end of each PCR to verify that a single DNA species was amplified. The following cycling parameters were used: start at 95°C for 5 min, denaturing at 95°C for 30 s, annealing at 60°C for 30 s, elongation at 72°C for 30 s, and a final 5 min extra extension at the end of the reaction to ensure that all amplicons were completely extended and repeated for 40 amplification cycles. The expression of test genes were normalized by using housekeeping gene i.e., 18S. The primer pairs used in the study are given in Table S1.

### ELISA Assays

HUVECs, LX2 cells and hepatoma cells (HBx-transfected and control-transfected both) were cultured with serum-free medium for 24 h. The supernatant was collected and concentrated on a Speed-Vac and ELISA for VEGF, PDGF-BB and TGF-β were performed using ELISA kits (Thermofisher Scientific) as per manufacturer's protocol. The optical density values were measured at 450 nm wavelength using microplate reader (Synergy/H1 Hybrid Multimode Plate Reader). Unknown values were extrapolated from the standard curves and normalized with respect to 10^6^ cells/ml (10).

### Immunophenotyping for Mesenchymal Genes

HBx-transfected, control-transfected, CD133 silenced and TGF-β stimulated hepatoma cells were fixed with permeabilizing solution containing Triton-X and ethyl alcohol for 20 min and incubated with primary antibodies CD133, VIM, and CDH1 followed by secondary antibody FITC, at 4°C in the dark for 45 min. After staining, the cells were analyzed using a flow cytometer (BD FACs Calibur). All experiments were performed at least in triplicates.

### Statistical Analysis

All quantitative data are expressed as mean ± standard deviation. Statistical significance is accepted as *p* ≤ 0.05(^*^), *p* ≤ 0.01(^**^), or *p* ≤ 0.001(^***^). Student's unpaired *t*-tests were used to analyze and compare the groups. All experiments were repeated at least three times.

## Results

### HBx-Transfected Cells Show Enhanced Invasion and Angiogenesis in Presence of Endothelial Cells

To assess the invasive properties of hepatoma cell lines in presence of HBV infection and stromal cells, we first conducted the transfection of Huh7 and HepG2 cells with the Hbx plasmids. After 48 h of transfection in hepatoma cells with pHBx-GFP, it was observed that Huh7 cells showed almost double the incorporation of the HBx plasmid as compared to that observed in HepG2 cells (Figures S1A–D). All experiments were henceforth conducted in Huh7 cells because they showed a higher transfection efficiency and HBx gene expression as compared to HepG2 cells (Figures S1E,F). The presence of gene expression of HBx in HBx-transfected Huh7 cells further validated the transfection (Figure S1F). Low gene expression of HBx was observed in HepG2 cells as compared to Huh7 (Figure S1F). Hence Huh7 cells were used for further assays.

**Figure 1:**
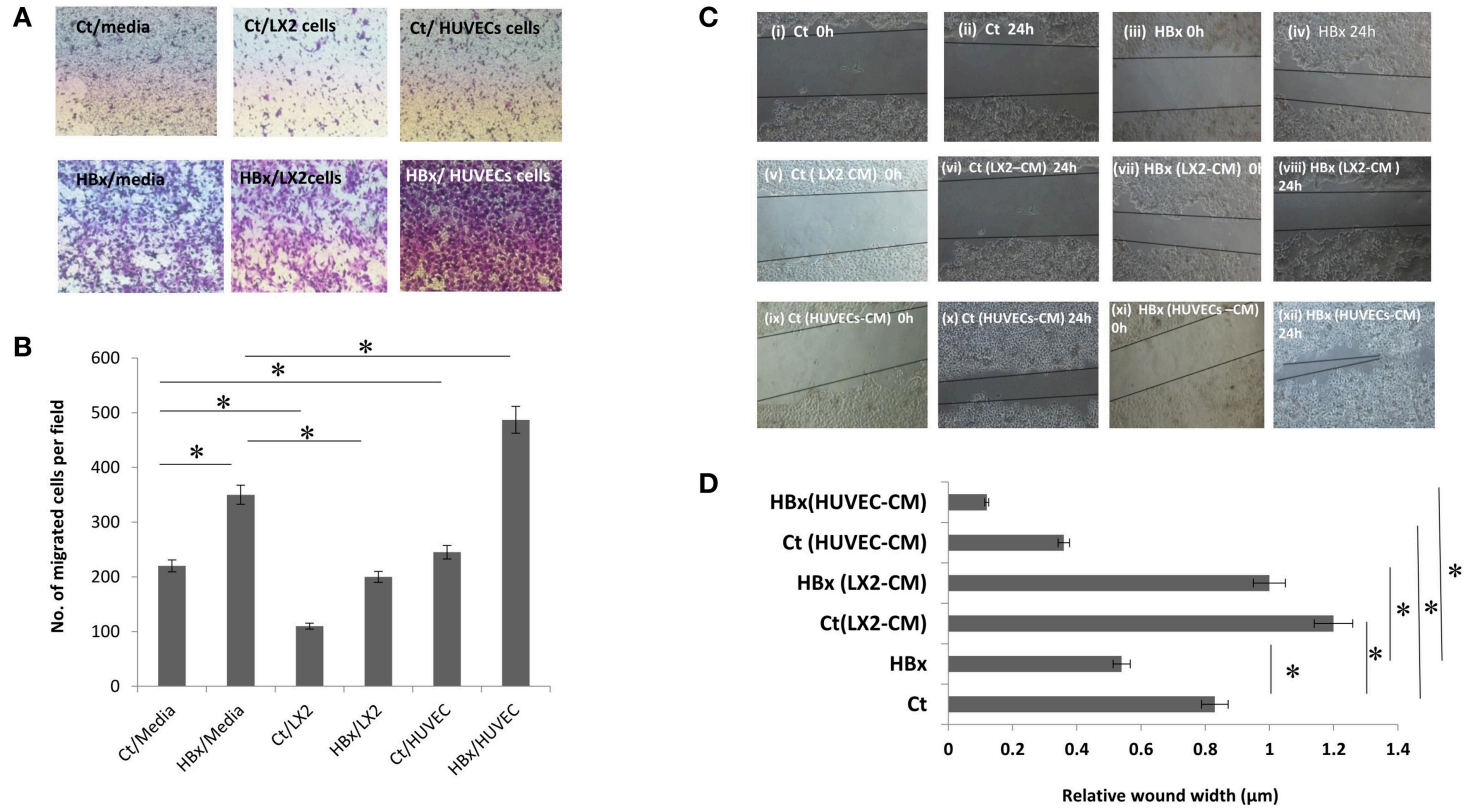
Functional assays showing migration of control transfected (Ct) and HBx transfected (HBx) Huh7 cells. **(A)** Phase contrast images of transwell assays showing migration of Ct and HBx cells from the upper chamber toward lower chamber containing media, LX2 cells or HUVECs after 24 h (magnification at 4X). **(B)** Bar diagram showing the number of migrated Ct and HBx cells in transwell assays under different conditions. **(C)** Phase contrast images showing migration of Ct and HBx cells after creation of a scratch/wound in presence of media alone, conditioned media (CM) from HUVECs (HUVECs-CM) or CM from LX2 cells (LX2-CM) at 0 and 24 h (magnification at 10X). **(D)** Bar diagram showing average relative wound width of Huh7 cells under different conditions. The relative wound width (μm) were calculated as wound width at time point 24 h divided by wound width at time point 0 h. Data is represented as mean ± SD (*n* = 3 each). *p < 0.05.

To study the effect of endothelial and stellate cells on the chemotactic properties of hepatoma cells with or without HBx, control or HBx-transfected Huh7 cells were cultured on the top of culture inserts while media, HUVECs or LX2 were placed beneath the inserts. After 24 h, in comparison to the control-transfected cells, we observed an increase in the number of HBx-transfected Huh7 cells that migrated in the lower chamber with media (Figures 1A,B). With LX2 cells in the lower chamber, the number of both HBx-transfected and control-cells that migrated toward the lower chamber substantially decreased as compared to the respective hepatoma cells with media (*P* < 0.05 each, Figures 1A,B). However, intriguingly, we observed a significant increase in the migration of HBx-transfected hepatoma cells toward HUVECs in comparison to that observed with media in the lower chamber (*P* < 0.05, Figures 1A,B, Figures S2C,D).

Next, to assess the cell migration, we performed wound healing migration assays of the control and HBx-transfected hepatoma cells in presence of media alone and CM from endothelial and stellate cells. Lesser wound width and early closure implied more migration of hepatoma cells. In presence of media, HBx-Huh7 cells showed decreased wound width in comparison to control Huh7 cells (Figures 1Ci–iv,D). After co-cultures with CM from LX2 cells, both control and HBx-Huh7 cells showed increased wound width as compared to that observed in these cells without co-cultures (Figures 1Cv–viii,D). In presence of CM from HUVECs, however, both control and HBx-Huh7 cells illustrated significantly reduced wound width showing maximum migration as compared to that observed in these cells under other conditions (Figures 1Cix–xii,D). Migration assays with Hep3B cells also showed decreased wound width in these cells when incubated with HUVECs CM as compared to that observed in these cells alone (Figures S2A,B).

### Upregulation of Mesenchymal Genes in HBx-Transfected Hepatoma Cells Co-cultured With Conditioned Media (CM) From Endothelial Cells

**Figure 2:**
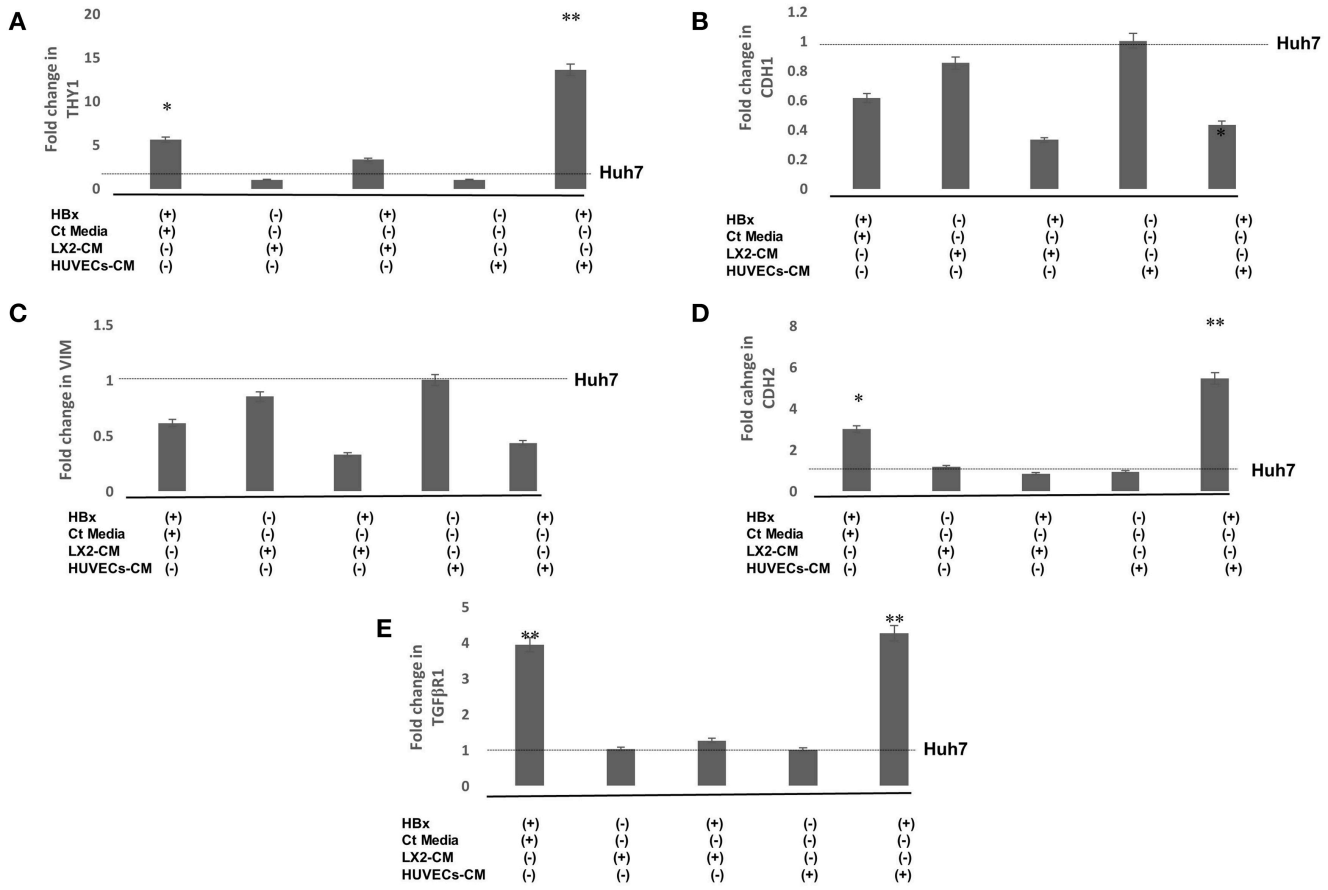
Relative mRNA expression of genes **(A)** THY-1 **(B)** CDH1 **(C)** VIM **(D)** CDH2 **(E)** TGFβR1 in Huh7 cells in presence of HBx, control (Ct) media, CM of LX2, and CM of HUVECs. Huh7 cells transfected with GFP plasmid were used as controls. Data is represented as mean ± SD (*n* = 3 each). *p < 0.05; **p < 0.01.

Epithelial to mesenchymal transition (EMT) in hepatoma cells was further validated by studying the expression of mesenchymal genes. RT-PCR studies showed that there was a significant increase in the expression of mesenchymal genes, Thy1, CDH2, and TGFβR1 in HBx-Huh7 cells as compared to that observed in the controls (Figures 2A,D,E). Gene expression of the studied mesenchymal genes in Huh7 cells co-cultured with LX2-CM were not significantly different as compared to the controls in presence or absence of HBx (Figures 2A–E). Expression of Thy1, CDH2, and TGFβR1 were markedly upregulated in HBx-Huh7 cells treated with HUVECs-CM in comparison to the controls (Figures 2A,C,D,E). Also, in presence of HUVECs-CM, the gene expression of epithelial marker, CDH1 was reduced in HBx-Huh7 cells as compared to the controls (Figure 2B).

### Endothelial Cells Secrete High Levels of TGF-β in the Cultures

**Figure 3:**
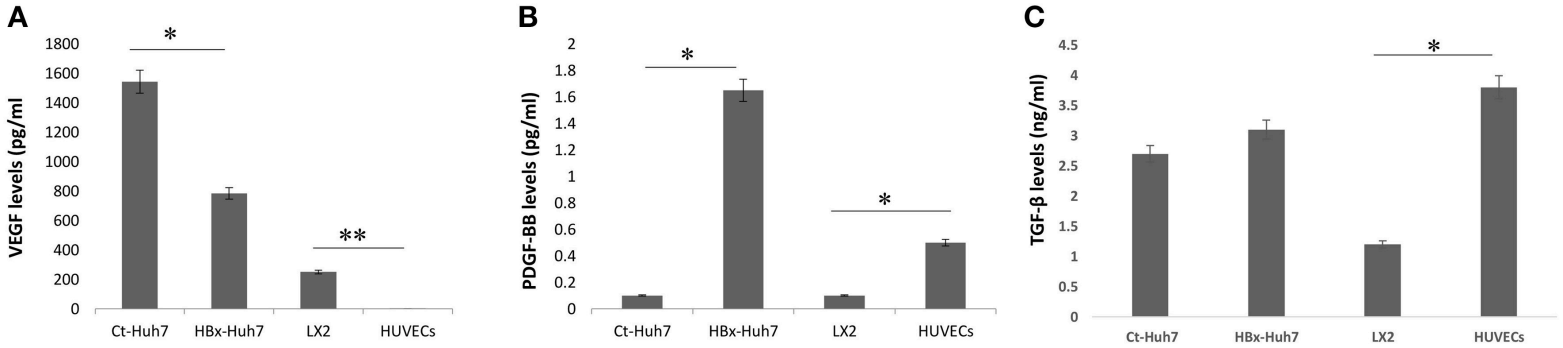
Bar diagrams showing the levels of **(A)** VEGF, **(B)** PDGF-BB, and **(C)** TGF-β secreted in culture supernatants or CM from control-transfected Huh7 (Ct-Huh7), HBx-transfected Huh7 (HBx-Huh7), LX2 cells, and HUVECs. Data is represented as mean ± SD (*n* = 3 each). *p < 0.05; **p < 0.01.

To investigate paracrine factors present in CM from endothelial and stellate cell lines, ELISAs for VEGF, PDGF-BB, and TGF-β were performed using supernatants from all the cells. Results illustrated that the basal levels of VEGF secreted by HUVECs were negligible. LX2 and hepatoma cells secreted increased amounts of VEGF in the cultures. In comparison to the HBx-Huh7 cells, Ct-Huh7 cells secreted higher amounts of VEGF in culture medium (*P* < 0.05, Figure 3A). In contrast, secretion of PDGF-BB was increased in HBx-Huh7 cells in comparison to Ct-Huh7 cells (*P* < 0.01, Figure 3B). TGF-β secretion was also enhanced in HBx-Huh7 cells and was maximum in HUVECs as compared to the Ct-Huh7 cells and LX2 cells (Figure 3C).

### TGF-β Imparts Invasive and Angiogenic Properties to Hepatoma Cells

**Figure 4:**
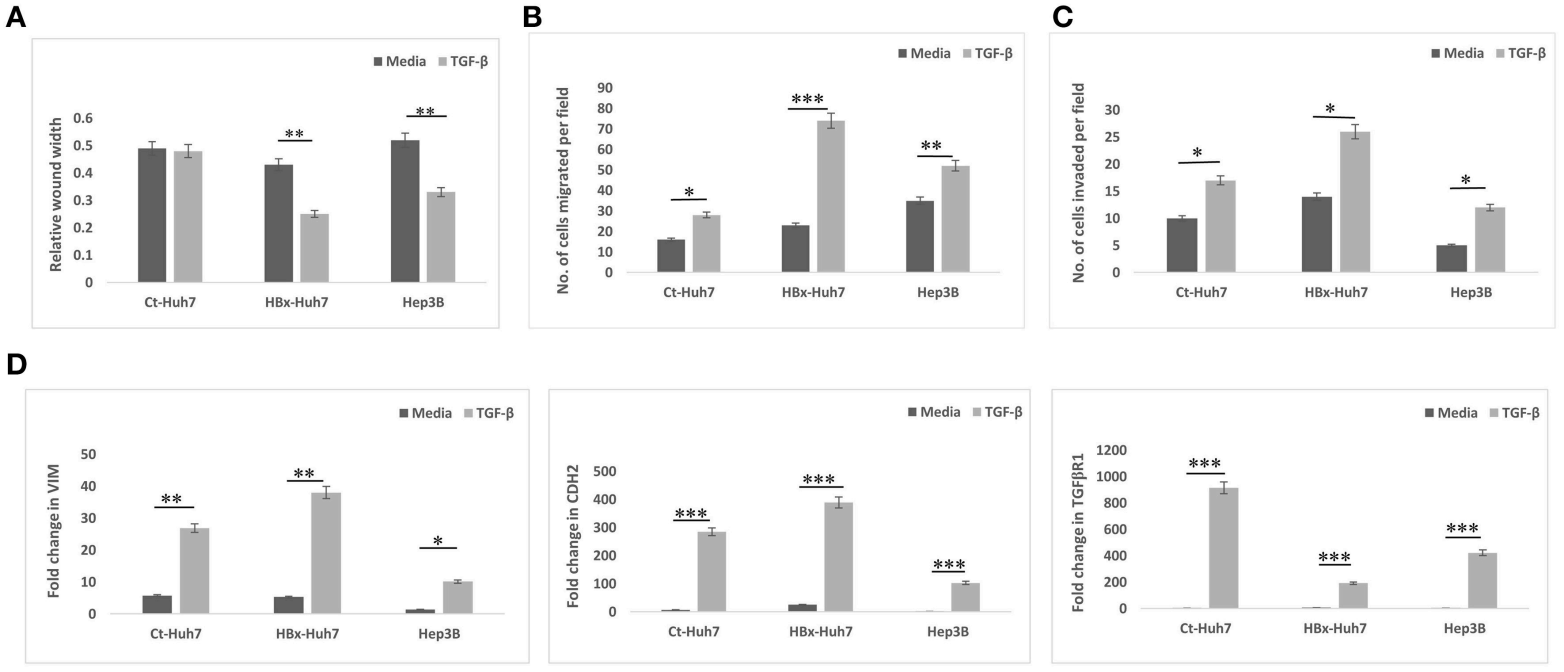
Epithelial mesenchymal transition in different hepatoma cells in presence of media alone or TGF-β. **(A)** Bar diagram showing average relative wound width at 24 h divided by that at 0 h in control transfected Huh7 (Ct-Huh7), HBx-Huh7, and Hep3B cells. **(B,C)** Chemotaxis and matrigel invasion transwell assays depicting migration of hepatoma cells from upper to lower chamber. **(D)** Relative mRNA expression of mesenchymal genes in hepatoma cells. Data is represented as mean ± SD (*n* = 3 each). *p < 0.05; **p < 0.01, ***p < 0.001.

To validate the role of TGF-β in HBx- induced EMT, further experiments were performed in presence of TGF-β. In these experiments, a positive control cell line with integrated HBV genome, Hep3B was also used along with Huh7 cells. In all cells, Ct-Huh7, HBx-Huh7, and Hep3B cells, TGF-β significantly enhanced mesenchymal properties including decreased wound width, increased chemotaxis (migration), and invasion (Figures 4A–C, Figures S3A–C). Maximum enhancement in mesenchymal properties and gene expression was observed in HBx-transfected Huh7 cells in presence of TGF- β (Figures 4A–C, Figures S3A–C). In terms of gene expression too, all cells including Huh7, HBx-Huh7, and Hep3B cells displayed significantly increased expression of mesenchymal genes, VIM, CDH2, and TGFβR1 in presence of TGF-β as compared to that observed in cells without TGF-β (Figure 4D).

### CD133 Mediates EMT in Hepatoma Cells

**Figure 5:**
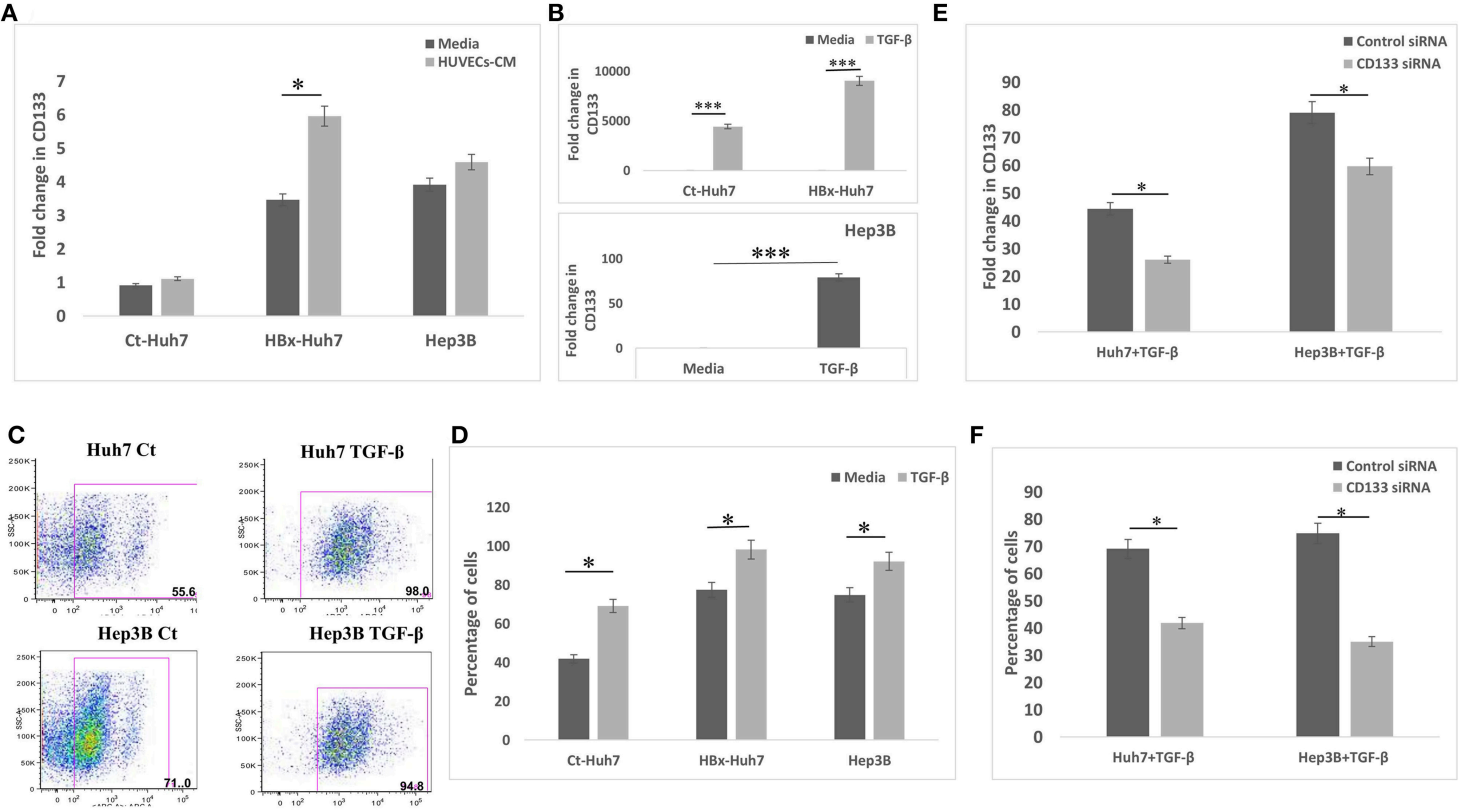
CD133 expression and silencing in different hepatoma cells. **(A)** Relative mRNA expression of CD133 in hepatoma cells in presence of media alone and CM of HUVECs. **(B)** Relative mRNA expression of CD133 in hepatoma cells in presence of media alone and TGF-β. **(C,D)** Dot-plots and bar diagram of flow cytometry data depicting percentage of CD133 positive cell population in Huh7 and Hep3B cells treated with media alone or TGF-β. **(E)** Relative mRNA expression of CD133 in TGF-β treated hepatoma cells after transfection with control or CD133 siRNA. **(F)** Flow cytometry quantitative data of CD133 gene in TGF-β treated hepatoma cells after transfection with control or CD133 siRNA. Data is represented as mean ± SD (*n* = 3 each). *p < 0.05; ***p < 0.001.

**Figure 6:**
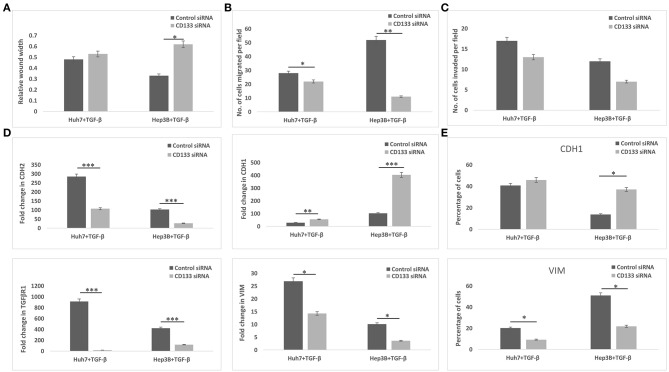
Epithelial mesenchymal transition in TGF-β incubated hepatoma cells treated with control siRNA or CD133 siRNA. **(A)** Bar diagram showing average of relative wound width at 24 h divided by that of 0 h. **(B,C)** Chemotaxis and matrigel invasion transwell assays showing migration of hepatoma cells from upper to lower chamber. Bar diagram showing the number of TGF-β exposed silenced hepatoma cells migrated toward the lower chamber in various conditions, respectively. **(D)** Relative mRNA expression of EMT genes in the hepatoma cells. **(E)** Flow cytometry analysis showing percentage of hepatoma cells expressing specific EMT markers (i.e., CDH1 and VIM). Data is represented as mean ± SD (*n* = 3 each). *p < 0.05; **p < 0.01; ***p < 0.001.

Since CD133 is known to regulate EMT in certain types of cancers, we hypothesized that CD133 may also be mediating EMT in HBx-transfected hepatoma cells in our study. CD133 expression was significantly high in HBx-Huh7 cells cocultured with HUVECs-CM as compared to that in control Huh7 cells (Figure 5A). CD133 gene expression was significantly enhanced in control Huh7, HBx-Huh7, and Hep3B cells when they were treated with TGF-β (Figure 5B). The protein expression of CD133 by flow cytometry assays further validated the above observations (Figures 5C,D). To elucidate the role of CD133 in EMT induction, we silenced CD133 by transfection with CD133 siRNA after TGF-β treatment in both Huh7 and Hep3B cells. HBx-Huh7 cells were not used in the CD133 siRNA experiments as double transfection led to significant cell death and loss. The inhibition of CD133 gene and protein expression in both the hepatoma cells was confirmed by RT-PCR and flow cytometery, respectively (Figures 5E,F, Figures S4A,B). In both the cell lines used, CD133 silencing led to a marked reduction of mesenchymal properties, including wound healing and chemotaxis as compared to control siRNA treated cells (Figures 6A–C, Figures S5A–C). The expression of mesenchymal genes, CDH2, VIM, TGFβR1 was substantially reduced and that of epithelial gene, CDH1 was increased in CD133 silenced as compared to control siRNA silenced cells (Figure 6D). Flow cytometry data also showed an increase in the percentage of CDH1 positive cells and a decrease in VIM positive cells after treatment with CD133 siRNA in comparison to that observed in the cells treated with control siRNA (Figure 6E).

## Discussion

A mutual crosstalk between cells and their microenvironment is essential for both normal tissue homeostasis and for tumor growth. Interactions between tumor cells and the tissue stroma strongly influence disease initiation, progression and ultimately patient survival. The major findings of the current study are that there exists a paracrine crosstalk between the liver tumor and the endothelial cells. Paracrine factor, TGF-β from endothelial cells imparts an invasive phenotype to the hepatoma cells via EMT and the presence of HBV protein, HBx in these cells further aggravate their invasive and mesenchymal properties.

Several studies report that endothelial cells are important regulators of tissue remodeling and endothelial cell-initiated signaling is known to promote the survival and tumorigenic potential of cancer stem cells (12). Secreted factors from endothelial cells have been reported to promote EMT and stemness traits in epithelial cells (13, 14). Studies by Chiew et al. have shown in co-culture models that physical supports from HepG2 cells are indispensable for the differentiation and remodeling of endothelial cells (15). In our study, we report that endothelial cells also stimulate migration and invasion of HCC cells via EMT in a paracrine manner even in the absence of a physical contact with the hepatoma cells. Intriguingly, tumor invasion properties and mesenchymal gene expression including increased expression of CD133, vimentin and N-cadherin and a decreased expression of E-cadherin are further enhanced in hepatoma cells in presence of both HUVECs and HBx. This indicates that factors from HUVECs act in conjunction with HBx-stimulated pathways to enhance tumor invasion. HBx has already been demonstrated to play a critical role in EMT in HCC (16). In contrast to HUVECs, we observed that fibroblastic LX2 cells significantly inhibited the migratory phenotype of both control and HBx-transfected cells in a contact-independent manner indicative of their tumor inhibitory role in HCC. Previous studies have reported that normal fibroblasts restrict the growth and progression of cancer in both contact dependent and independent manner (17). In an altered tumor environment *in vivo*, the restrictive tumor fibroblasts can also turn into supportive fibroblast as shown existence of cancer—associated fibroblasts (CAFs) in HCCs. Secreted factors from CAFs are known to support the growth of Hep3B cells via increased production of cytokines such as hepatocyte growth factor (HGF), FGF, IL-6, SDF-1 (18). We observed an inhibitory function of LX2 cells in our cultures as these were normal fibroblasts and not CAFs.

Analysis of paracrine factors from different cell types revealed that VEGF secretion was more in supernatants from control hepatoma cells as compared to that observed in HBx-transfected cells while PDGF-BB and TGF-β levels were markedly enhanced in HBx-transfected hepatoma cells indicating that increased VEGF secretion may be inhibiting EMT while PDGF-BB and TGF-β may be a promoter of EMT phenotype. A study by Hong et al. have reported an inhibitory role of VEGF in EMT, however there are other contrasting studies which have shown that VEGF connects EMT and angiogenesis in tumor progression (19–21). The role of PDGF in promoting TGF-β mediated EMT and hence tumor invasion is well reported (22). We focussed our attention on TGF-β, as its secretion was highest in supernatants from HUVECs. A concentration of 5 ng/ml TGF-β was selected as HUVECs secreted TGF-β in the range of 3–5 ng/ml of TGF**-**β levels. TGF-β induces EMT in several cancer cell lines by acquisition of mesenchymal morphology and increased expression of vimentin and Thy-1 (23, 24). In our system too, TGF-β promoted mesenchymal characteristics in all the cell lines including control Huh7, HBx-transfected Huh7 and Hep3B cells.

EMT is one of the early events in primary tumor invasion during which tumor cells lose epithelial markers and gain mesenchymal traits that confer stem-like properties and a migratory phenotype (23, 25). TGF-β has been earlier known to induce cancer stemness and EMT via CD133 (25, 26). HBx mice have also been shown to also exhibit a stronger expression of CD133 previously (11). Hence, it was worthwhile to further investigate if CD133 also connects endothelial cell-mediated increase in tumor invasive properties of HBx-transfected hepatoma cells. We observed a significant increase in CD133 expression in HBx-transfected Huh7 cells both after treatments with CM from HUVECs as well as with TGF-β. After the silencing of CD133 gene in TGF-β treated hepatoma cells, there was a dramatic reduction in mesenchymal properties as well as an increment in epithelial characteristics, clearly indicating that the gain of mesenchymal features in these cells is attributed to increase in the expression of stemness-associated marker, CD133.

In summary, our study describes that increased expression of HBx during HBV infection in association with secretory factors released by HBx-transfected hepatoma cells or neighboring endothelial cells, particularly TGF-β, imparts invasive properties to these cells via EMT induction through increased expression of CD133. It would be worthwhile to validate these findings under *in vivo* settings where the tumor microenvironment is much more complex, as macrophages, neutrophils, and lymphocytes are also recruited to the tumor stroma. Most therapeutic strategies against cancer have focused either on targeting tumor cells or the stromal cells, including various angiogenesis inhibitors, both of which have limited benefits. Given the vital role of tumor-stroma dialogue in tumor metastasis, it becomes imperative to identify the underlying signaling mechanisms of interaction and designing of novel therapeutic strategies to manipulate these interactions for blocking tumor growth and progression.

## Author Contributions

PR performed all cell culture experiments, migration, chemotaxis, invasion, immunophenotyping and ELISA assays, collected and analyzed all data and drafted the manuscript. HS helped in performing the functional assays. MH helped in standardizing the initial transfection experiments. MC and NT helped in flow cytometry assays. VN and DT helped PR in the editing of the manuscript. SK got the funding for the study, designed the study, performed data analysis along with PR and finalized the manuscript.

### Conflict of Interest Statement

The authors declare that the research was conducted in the absence of any commercial or financial relationships that could be construed as a potential conflict of interest.

## References

[1] Ferlay J Shin HR Bray F Forman D Mathers C Parkin DM. Estimates of worldwide burden of cancer in 2008, GLOBOCAN 2008. Int J Cancer. (2010) 127:2893–917. 10.1002/ijc.25516 21351269

[2] Beasley RP Hwang LY Lin CC Chien CS. Hepatocellular carcinoma and hepatitis B virus. A prospective study of 22707 men in Taiwan. Lancet. (1981) 2:1129–33. 10.1016/S0140-6736(81)90585-7 6118576

[3] Kidd-Ljunggren K Oberg M Kidd AH. The hepatitis B virus X gene: analysis of functional domain variation and gene phylogeny using multiple sequences. J Gen Virol. (1995) 76 (Pt 9):2119–30. 10.1099/0022-1317-76-9-2119 7561749

[4] Feitelson MA Lee J. Hepatitis B virus integration, fragile sites, and hepatocarcinogenesis. Cancer Lett. (2007) 252:157–70. 10.1016/j.canlet.2006.11.010 17188425

[5] Zhu M Guo J Li W Xia H Lu Y Dong X. HBx induced AFP receptor expressed to activate PI3K/AKT signal to promote expression of Src in liver cells and hepatoma. BMC Cancer. (2015) 15:362. 10.1186/s12885-015-1384-9 25943101 PMC4427932

[6] Heindryckx F Gerwins P. Targeting the tumor stroma in hepatocellular carcinoma. World J Hepatol. (2015) 7:165–76. 10.4254/wjh.v7.i2.165 25729472 PMC4342599

[7] Folkman J. Role of angiogenesis in tumor growth and metastasis. Semin Oncol. (2002) 29:15–8. 10.1053/sonc.2002.37263 12516034

[8] Fu S Zhou R Li N Huang Y Fan XG Hepatitis B virus X protein in liver tumor microenvironment. Tumor Biol. (2016) 37:15371–81. 10.1007/s13277-016-5406-2 PMC5250643 27658781

[9] Khodarev NN Yu J Labay E Darga T Brown CK Mauceri HJ. Tumour-endothelium interactions in co-culture: coordinated changes of gene expression profiles and phenotypic properties of endothelial cells. J Cell Sci. (2002) 116:1013–22. 10.1242/jcs.00281 12584245

[10] Ding S Chen G Zhang W Xing C Xu X Xie H. MRC-5 fibroblast-conditioned medium influences multiple pathways regulating invasion, migration, proliferation, and apoptosis in hepatocellular carcinoma. J Transl Med. (2015) 13:237. 10.1186/s12967-015-0588-8 26198300 PMC4508812

[11] Rawat S Bouchard MJ. The hepatitis B virus (HBV) HBx protein activates AKT to simultaneously regulate HBV replication and hepatocyte survival. J Virol. (2014) 89:999 −1012. 10.1128/JVI.02440-14 25355887 PMC4300660

[12] Selek L Dhobb M Sanden BV Berger F Wion D. Existence of tumor-derived endothelial cells suggests an additional role for endothelial-to-mesenchymal transition in tumor progression. Int J Cancer. (2011) 128:1502–3. 10.1002/ijc.25446 20473934

[13] Zhang Z Dong Z Lauxen IS Filho MSA Nor JE. Endothelial cell-secreted EGF induces epithelial to mesenchymal transition and endows head and neck cancer cells with stem-like phenotype. Cancer Res. (2014) 74:2869–81. 10.1158/0008-5472.CAN-13-2032 24686166 PMC4028029

[14] Sigurdsson V Hilmarsdottir B Sigmundsdottir H Fridriksdottir AJR Ringner M. Endothelial induced EMT in breast epithelial cells with stem cell properties. PLoS ONE. (2011) 6:e23833. 10.1371/journal.pone.0023833 21915264 PMC3167828

[15] Chiew GGY Fu A Low KP Luo KQ. Physical supports from liver cancer cells are essential for differentiation and remodeling of endothelial cells in a HepG2-HUVEC co-culture model. Sci. Rep. (2015) 5:10801. 10.1038/srep10801 26053957 PMC4459107

[16] Jin Y Wu D Yang W Weng M Li Y Wang X. Hepatitis B virus x protein induces epithelial-mesenchymal transition of hepatocellular carcinoma cells by regulating long non-coding RNA. Virol J. (2017) 14:238. 10.1186/s12985-017-0903-5 29258558 PMC5735895

[17] Alkasaliasa T Flaberga E Kashubaa V Alexeyenkoa A Pavlovaa T Savchenkoa A Inhibition of tumor cell proliferation and motility by fibroblasts is both contact and soluble factor dependent. Proc Natl Acad Sci USA. (2014) 111:17188–93. 10.1073/pnas.1419554111 25404301 PMC4260581

[18] Chen HY Chen ZX Huang RF Lin N Wang XZ. Hepatitis B virus X protein activates human hepatic stellate cells through upregulating TGFβ1. Genet Mol Res. (2014) 13:8645–56. 10.4238/2014.October.27.4 25366754

[19] Hong JP Li XM Li MX Zheng FL VEGF suppresses epithelial-mesenchymal transition by inhibiting the expression of Smad3 and miR-192, a Smad3-dependent microRNA. Int J Mol Med. (2013) 31:1436–42. 10.3892/ijmm.2013.1337 23588932

[20] Lu Y Qin T Li J Wang L Zhang Q Jiang Z. MicroRNA-140-5p inhibits invasion and angiogenesis through targeting VEGF-A in breast cancer. Cancer Gene Ther. (2017) 24:386–92. 10.1038/cgt.2017.30 28752859 PMC5668497

[21] Lou W Liu J Gao Y Zhong G Chen D Shen J MicroRNAs in cancer metastasis and angiogenesis. Oncotarget. (2017) 70:115787–802. 10.18632/oncotarget.23115 PMC5777813 29383201

[22] Heldin CH. Targeting the PDGF signaling pathway in tumor treatment. Cell Commun Signal. (2013) 11:97. 10.1186/1478-811X-11-97 24359404 PMC3878225

[23] Pirozzi G Tirino V Camerlingo R Franco R La Rocca A Liguori E Epithelial to mesenchymal transition by TGFb-1 induction increases stemness characteristics in primary non-small cell lung cancer cell line. PLoS ONE. (2011) 6:e21548 10.1371/journal.pone.0021548 21738704 PMC3128060

[24] Vedagiri D Lashkari HV Mangani AS Kumar JM Jose J Thatipalli AR. An atypical system for studying epithelial-mesenchymal transition in hepatocellular carcinoma. Sci. Rep. (2016) 6:26282. 10.1038/srep26282 27197891 PMC4873837

[25] Mani SA Guo W Liao MJ Eaton EN Ayyanan A Zhou AY. The epithelial-mesenchymal transition generates cells with properties of stem cells. Cell. (2008) 133:704–15. 10.1016/j.cell.2008.03.027 18485877 PMC2728032

[26] Sowa T Menju T Sonobe M Nakanishi T Shikuma K Imamura N. Association between epithelial-mesenchymal transition and cancer stemness and their effect on the prognosis of lung adenocarcinoma. Cancer Med. (2015) 4:1853–62. 10.1002/cam4.556 26471868 PMC5123719

