# Supplementary material for: Endothelial Cell-Derived TGF-β Promotes Epithelial-Mesenchymal Transition via CD133 in HBx-Infected Hepatoma Cells
Source: Front Oncol. 2019 Apr 24;9:308. doi: 10.3389/fonc.2019.00308 (PMC6491671; doi:10.3389/fonc.2019.00308)
Supplement: Supplementary file 1 [file Table_1.pdf]

Table S1: List of Forward and Reverse primer sequences used for the analysis of mRNA expression in the study..

S.No	GENE NAME	FORWARD SEQUENCE	REVERSE SEQUENCE
1.	18 s	5’ GTAACCCGTTGAACCCCATT 3’	5’ CCATCCAATCGGTAGTAGCG 3’
2.	Thy-1	5’GCTGCTTCTGTCTGGTTTAT 3’	5’ ACTCCTCATCCAAGTCTCTC 3’
3.	VIM	5’CAGATGCGTGAAATGGAAGA 3’	5’TGGAAGAGGCAGAGAAATCC 3’
4..	CDH1	5’CCCCCTGTTGGTGTCTTTATT 3’	5’ATTCGGGCTTGTTGTCATTC 3’
5.	CDH2	5’GCTCCCTTAATTCCTCAAGTAG 3’	5’TCATCACCTCCACCATACA 3’
6.	CD133	5’ CCACATGCTACATCCAGATAC 3’	5’CCAGGACACAGCATAGAATAAT 3’
7.	TGFβR1	5’GCTGTATTGCAGACTTAGGACT 3’	5’TTTTTGTTCCCACTCTGTGGTT 3’
