# Supplementary material for: Endothelial Cell-Derived TGF-β Promotes Epithelial-Mesenchymal Transition via CD133 in HBx-Infected Hepatoma Cells
Source: Front Oncol. 2019 Apr 24;9:308. doi: 10.3389/fonc.2019.00308 (PMC6491671; doi:10.3389/fonc.2019.00308)
Supplement: Supplementary file 2 [file Data_Sheet_1.PDF]

Figure S1

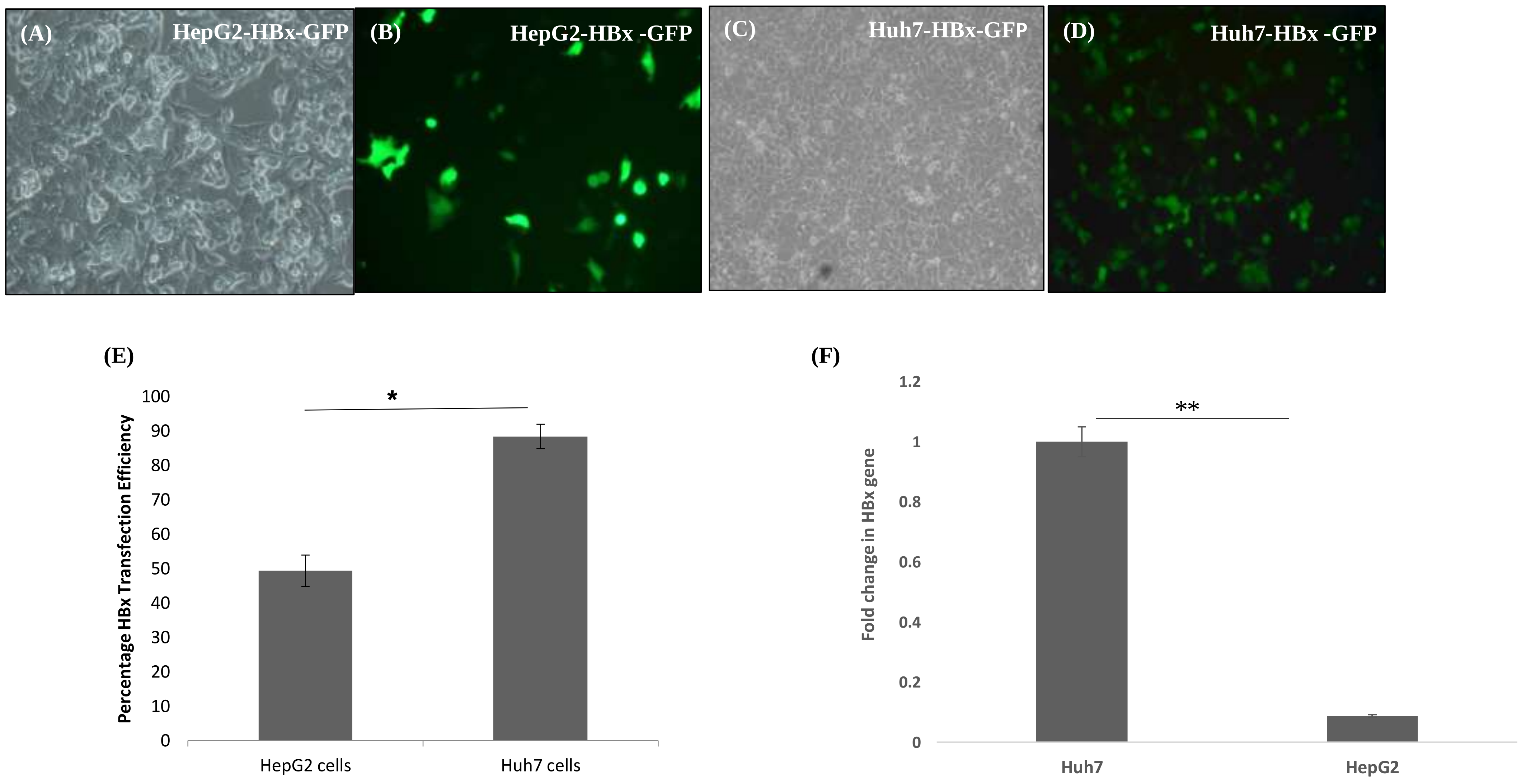

(A)
HepG2-HBx-GFP
(B)
HepG2-HBx -GFP
(C)
Huh7-HBx-GFP
(D)
Huh7-HBx -GFP
(E)
Percentage HBx Transfection Efficiency
100
90
80
70
60
50
40
30
20
10
0
*
HepG2 cells
Huh7 cells
(F)
Fold change in HBx gene
1.2
1
0.8
0.6
0.4
0.2
0
**
Huh7
HepG2

Figure S2

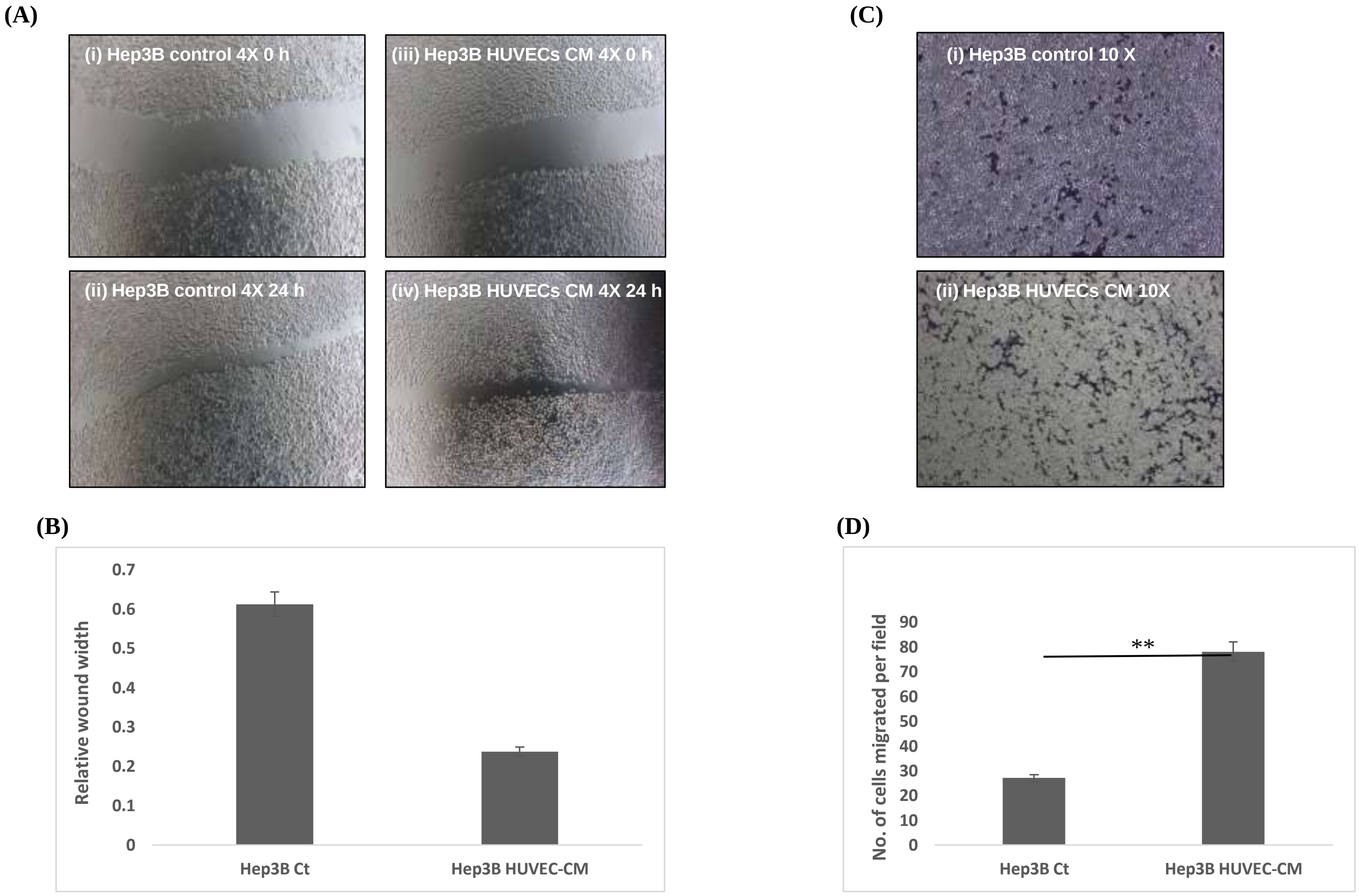
(A)
(i) Hep3B control 4X 0 h
(iii) Hep3B HUVECs CM 4X 0 h
(ii) Hep3B control 4X 24 h
(iv) Hep3B HUVECs CM 4X 24 h
(B)
Relative wound width
0.7
0.6
0.5
0.4
0.3
0.2
0.1
0
Hep3B Ct
Hep3B HUVEC-CM
(C)
(i) Hep3B control 10 X
(ii) Hep3B HUVECs CM 10X
(D)
No. of cells migrated per field
90
80
70
60
50
40
30
20
10
0
**
Hep3B Ct
Hep3B HUVEC-CM

Figure S3

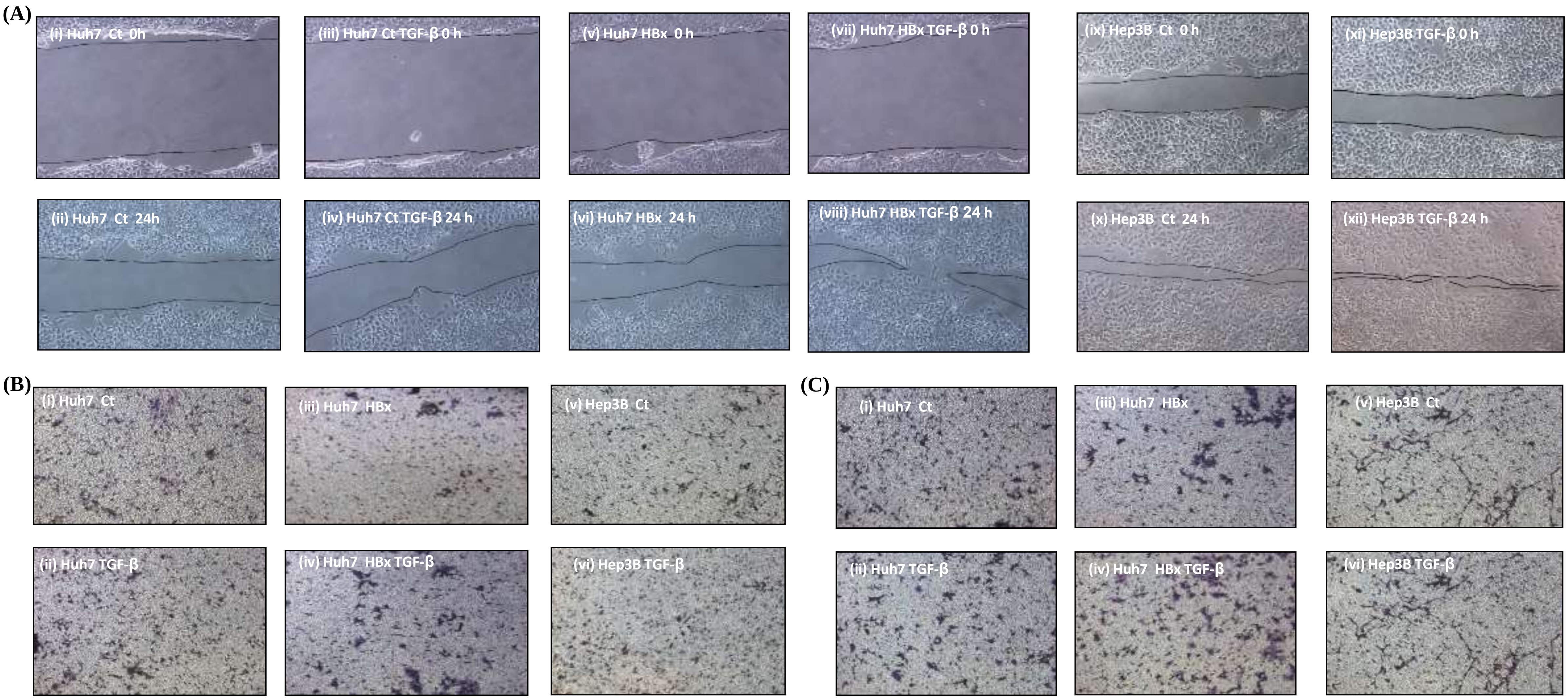
(A)
(i) Huh7 Ct 0h
(iii) Huh7 Ct TGF-β 0 h
(v) Huh7 HBx 0 h
(vii) Huh7 HBx TGF-β 0 h
(ix) Hep3B Ct 0 h
(xi) Hep3B TGF-β 0 h
(ii) Huh7 Ct 24h
(iv) Huh7 Ct TGF-β 24 h
(vi) Huh7 HBx 24 h
(viii) Huh7 HBx TGF-β 24 h
(x) Hep3B Ct 24 h
(xii) Hep3B TGF-β 24 h
(B)
(i) Huh7 Ct
(iii) Huh7 HBx
(v) Hep3B Ct
(ii) Huh7 TGF-β
(iv) Huh7 HBx TGF-β
(vi) Hep3B TGF-β
(C)
(i) Huh7 Ct
(iii) Huh7 HBx
(v) Hep3B Ct
(ii) Huh7 TGF-β
(iv) Huh7 HBx TGF-β
(vi) Hep3B TGF-β

Figure S4

(A)

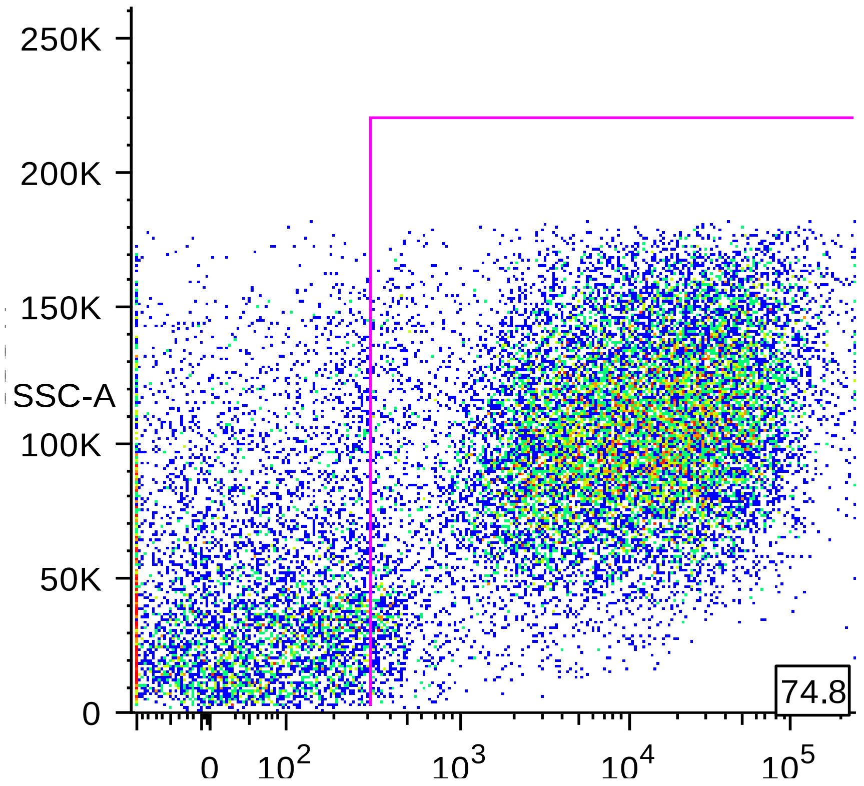

Huh7 cells control siRNA transfected

(B)

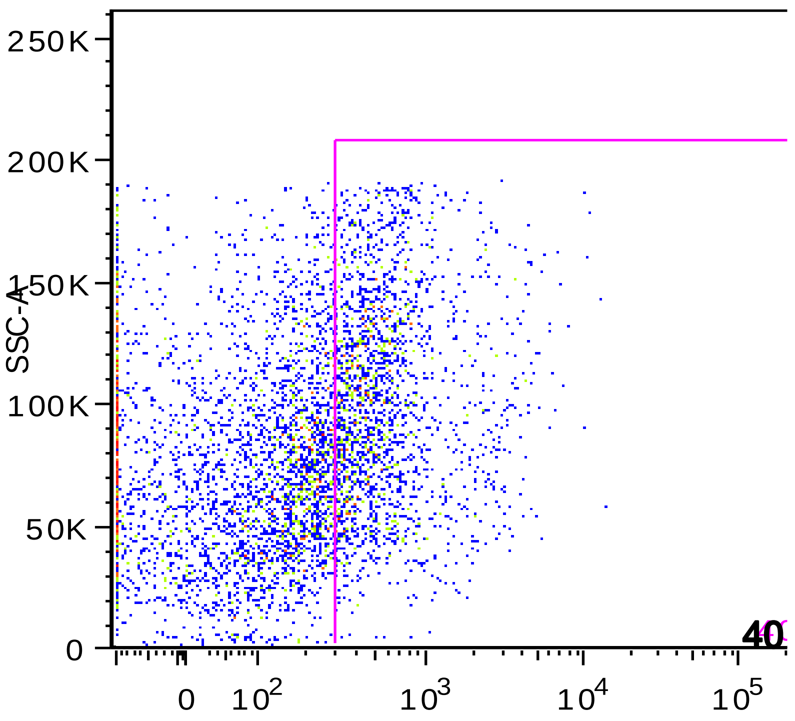

Huh7 cells CD133 siRNA transfected

Figure S5

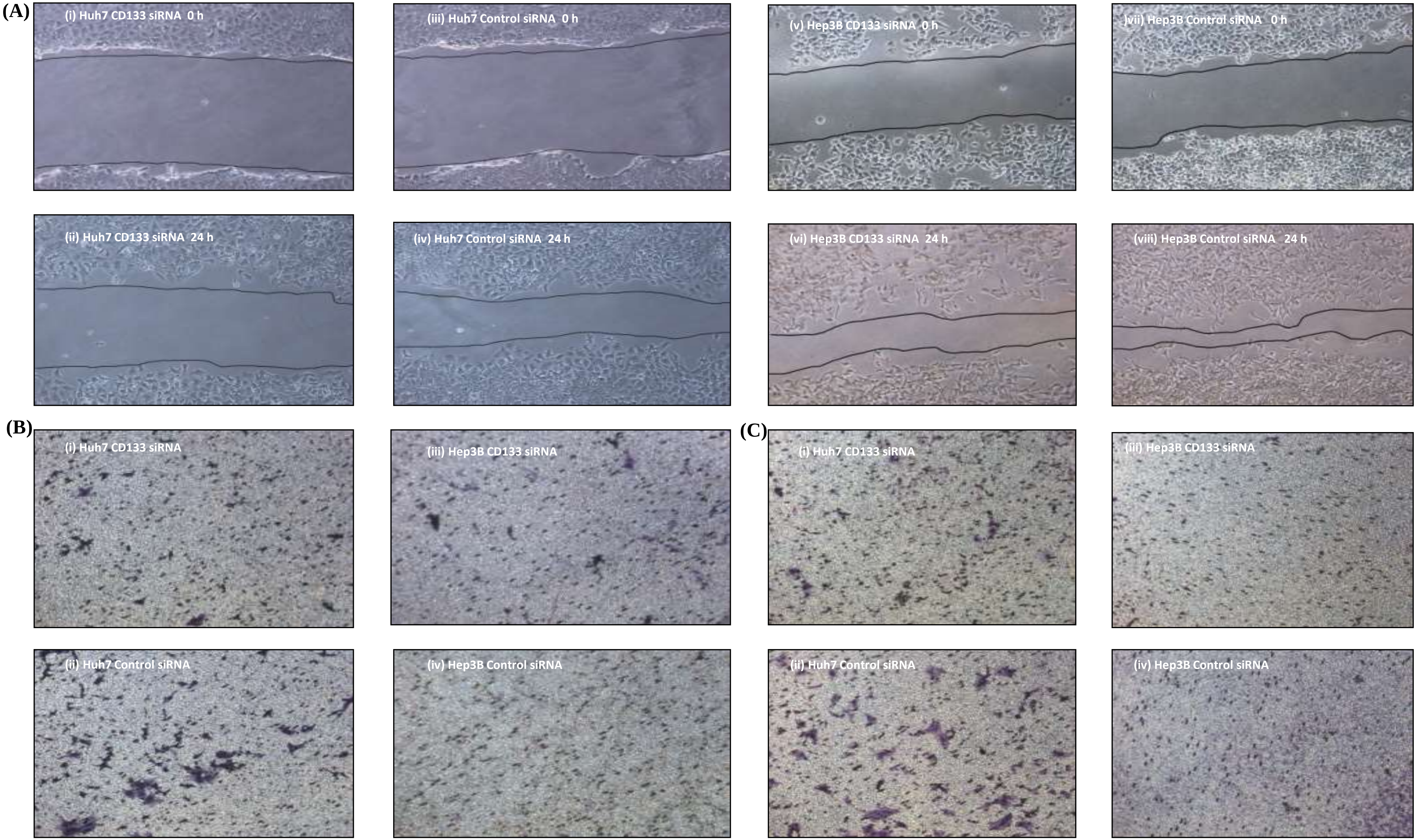
(A)
(i) Huh7 CD133 siRNA 0 h
(iii) Huh7 Control siRNA 0 h
(v) Hep3B CD133 siRNA 0 h
(vii) Hep3B Control siRNA 0 h
(ii) Huh7 CD133 siRNA 24 h
(iv) Huh7 Control siRNA 24 h
(vi) Hep3B CD133 siRNA 24 h
(viii) Hep3B Control siRNA 24 h
(B)
(i) Huh7 CD133 siRNA
(iii) Hep3B CD133 siRNA
(ii) Huh7 Control siRNA
(iv) Hep3B Control siRNA
(C)
(i) Huh7 CD133 siRNA
(iii) Hep3B CD133 siRNA
(ii) Huh7 Control siRNA
(iv) Hep3B Control siRNA
