# Supplementary material for: Endothelial Cell-Derived TGF-β Promotes Epithelial-Mesenchymal Transition via CD133 in HBx-Infected Hepatoma Cells
Source: Front Oncol. 2019 Apr 24;9:308. doi: 10.3389/fonc.2019.00308 (PMC6491671; doi:10.3389/fonc.2019.00308)
Supplement: Supplementary file 3 [file Data_Sheet_2.pdf]

Supplementary figures legends:

Figure S1: Transfection of hepatoma cells with HBx. (A) and (B) Phase contrast and flourescence images of HepG2 cells after 48h of transfection with HBx-GFP (C) and (D) Phase contrast and flourescence images of Huh7 cells after 48h of transfection with HBx-GFP (E) Bar diagram showing average percentage of transfection efficiency of the hepatoma cells with HBx. (F) HBx expression in HBx-transfected Huh7 and HepG2 cells by Real time analysis. Data is represented as mean ± SD. (n= 3 each). *p< 0.05; **p<0.01 .

Figure S2: (A) Phase contrast images of Hep3B cells treated with media alone or conditioned media (CM) from HUVECs at 0 and 24 h (magnification at 4X) (B) Bar diagram showing average of relative wound width at 24 h divided by that at 0 h. (C): Phase contrast images of chemotaxis transwell assays showing migration of hepatoma cells (Hep3B) from upper to lower chamber containing media or CM from HUVECs (magnification at 10X) (D) Bar diagram showing the number of hepatoma cells that migrated towards the lower chamber. Data is represented as mean ± SD. (n= 3 each). **p<0.01.

Figure S3: (A) Phase contrast images of wound healing scratch assays of Huh7 and Hep3B cells with media alone or TGF-β at 0 and 24 h (magnification at 10X). (B) and (C) Phase contrast images of chemotaxis transwell and matrigel invasion assays showing migration and invasion of hepatoma cells from upper to lower chamber containing media alone or TGF-β (magnification at 4X).

Figure S4: Flow cytometry dot plot of CD133 gene expression in (A) Huh7 cells transfected with control siRNA and (B) Huh7 cells transfected with CD133 siRNA.

Figure S5: (A) Phase contrast images of wound healing scratch assays of TGF-β activated control and CD133 siRNA silenced Huh7 and Hep3B cells at 0 and 24 h (magnification at 10X). (B) and (C) Phase contrast images of invasion and chemotaxis transwell assays showing migration and invasion of control and CD133 siRNA silenced hepatoma cells (magnification at 4X) .
